# Naturally occurring anti-glycan antibodies binding to Globo H-expressing cells identify ovarian cancer patients

**DOI:** 10.1186/s13048-017-0305-8

**Published:** 2017-02-10

**Authors:** Tatiana Pochechueva, Shahidul Alam, Andreas Schötzau, Alexander Chinarev, Nicolai V. Bovin, Neville F. Hacker, Francis Jacob, Viola Heinzelmann-Schwarz

**Affiliations:** 1 Ovarian Cancer Research, Department of Biomedicine, University Hospital Basel, University of Basel, Basel, Switzerland; 2 Glyco-Oncology, Ovarian Cancer Research, Department of Biomedicine, University Hospital Basel, University of Basel, Basel, Switzerland; 3 Shemyakin- Ovchinnikov Institute of Bioorganic Chemistry, Russian Academy of Sciences, 117997 Moscow, Russian Federation; 4 Royal Hospital for Women, Gynecological Cancer Centre, School of Women’s and Children’s Health, University of New South Wales, Sydney, Australia; 5 Hospital for Women, Department of Gynecology and Gynaecological Oncology, University Hospital Basel, Basel, Switzerland

**Keywords:** Ovarian cancer, Glycosphingolipids, Suspension array, Glycan

## Abstract

**Background:**

Glycosphingolipids are important compounds of the plasma membrane of mammalian cells and a number of them have been associated with malignant transformation and progression, reinforcing tumour aggressiveness and metastasis. Here we investigated the levels of naturally occurring anti-glycan antibodies to Globo H in blood plasma obtained from high-grade serous ovarian cancer patients (SOC) and women without gynaecological malignancies (control) using suspension glycan array technology employing chemically synthesized glycans as antibody targets.

**Results:**

We found that anti-human Globo H IgG antibodies were able to significantly discriminate SOC from controls (*P* < 0.05). A combination with the clinically used tumour marker CA125 increased the diagnostic performance (AUC 0.8711). We next compared suspension array with standard flow cytometry in plasma samples and found that the level of anti-Globo H antibodies highly correlated (*r* = 0.992). The incubation of plasma-derived anti-glycan antibodies with chemically synthesized (presented on fluorescence microspheres) and native Globo H (expressed on Globo H-positive cell lines) revealed strong reactivity of naturally occurring human anti-Globo H antibodies towards its antigen expressed on ovarian cancer cells.

**Conclusions:**

Our data demonstrate that human plasma-derived antibodies to Globo H as well as the presence of the antigen might be considered as therapeutic option in ovarian cancer.

## Background

Globo H is a glycosphingolipid of the globo series with a sugar terminus resembling the blood group antigen H determinant. First identified in human teratocarcinoma and breast cancer cells [1, 2], Globo H was found to be overexpressed on the cell surface of several epithelial cancers such as breast, colon, endometrial, gastric, pancreatic, lung, and prostate cancers [3–5]. It is also moderately expressed in normal epithelial tissues (lung, breast, prostate, stomach, pancreas, and ovary), but its distribution is restricted to apical epithelial cells at lumen borders [5]. Functionally, Globo H has been associated with tumour stem cells [6], to be a potent inducer of angiogenesis [7], and an immunosuppressor through Notch signalling [8]. The high Globo H expression by only cancer and cancer stem cells made it an attractive target for generation of therapeutic cancer vaccines. These vaccines underwent the long history of development and improvement and are currently being tested in clinical trials for treatment [9–11]. Globo H has therefore been considered as one promising tumor-associated glycan biomarker, in particular for breast cancer.

Plasma-derived naturally occurring anti-glycan antibodies (AGA) have been associated to various human diseases including cystic fibrosis [12], Crohn’s disease [13], and various malignancies [14–18]. Also healthy humans harbour sets of AGA to blood group-, xeno- (heterophil), and infection-related glycan motifs [19]. Globo H was also shown to be an antigen for naturally occurring plasma-derived AGA in breast and some other epithelial cancers with high specificity [20]. These antibodies were assessed by the glycan microarray technology and their levels were significantly different in serum of breast cancer patients and healthy individuals [21]. Despite these few reports, there is limited knowledge on Globo H antigen expression on cell lines and the presence of naturally circulating AGA in plasma from ovarian cancer patients.

We assessed the levels of anti-Globo H antibodies of IgG and IgM isotypes in healthy and serous ovarian cancer (SOC) patients. We used our “in house” developed suspension glycan array (SGA), a robust tool for AGA profiling [15, 22], to detect anti-Globo H antibodies in human plasma samples for discrimination of ovarian cancer patients and normal controls. We also compared SGA and standard flow cytometry in order to address the following questions; a) whether AGA levels are comparable in both methods, and b) whether those antibodies can recognize native Globo H epitope on the cell surface of cancer cell lines.

## Methods

### Human subjects

Two groups of female donors were involved in the current study. All patients were over the age of 18 and were prospectively included after giving informed consent in accordance with ethical regulations (Hunter Area Research Ethics 04/04/07/3.04; South Eastern Sydney Illawarra HREC/AURED Ref:08/09/17/3.02). The serous ovarian cancer (SOC) group consisted from patients with serous ovarian cancer with Grade 2 and 3 of all FIGO stages (SOC *n* = 19). The control group (*n* = 29) consisted of women without macroscopically confirmed gynaecological malignancy including comprising of benign donors, patients with benign gynaecological tumours (leiomyoma, cystadenoma, or fibroadenoma) and patients undergoing medical control due to previous family history of breast cancer or bearing known *BRCA* mutations. Women in the control group had no other type of malignancy at the date of collection. Patients were either admitted with an adnexal mass to the Gynaecological Cancer Centre of the Royal Hospital for Women, Randwick, Australia or were seen as outpatients to the Hereditary Cancer Centre of The Prince of Wales Hospital, Randwick, Australia. The processing of blood plasma samples was performed as previously described [15, 23].

### Suspension glycan array

Glyco-polymers with end-biotin group*-* Sialylated and sulfated glycans were chemically synthesised (Lectinity Holdings, Moscow, Russia. The glycopolymers, Glyc(20)–PAA–biot_1_, used for coupling to fluorescent beads were produced in-house as previously described [24]. The glycopolymers are composed of a polyacrylamide carrier (PAA, number of the average polymerisation degree, *n* = 220) provided with end biotin groups and side-pendant Glyc residues, that are statistically distributed along the polymer backbone. The content of monomer units with glycan substitution is 20 mol %. Non-glycosylated monomer units are substituted with an ethanolamine residue for the regular Glyc(20)–PAA–biot_1_. GD_3_ and GM_2_ glycans were purchased from Elicityl (Crolles, France) and then used for synthesis of Glyc(20)–PAA–biot_1_, as described above.

Modification of fluorescent microbeads*-* Biotinylated glycopolymers were coupled to fluorescent Bio-Plex Pro™ Magnetic COOH beads of 6.5 μm diameter with distinct spectral “addresses” (Bio-Rad Laboratories Inc., Cressier, Switzerland). Each bead’s region was embedded with a precise ratio of red and infrared fluorescent dyes allowing its identification using Bio-Plex 200 Suspension Array System (Bio-Rad Laboratories Inc., Hercules, CA, USA). Coupling of biotinylated glycopolymers using biot–PEG_23_–NH_2_ (Hetero-bifunctional PEG, MW = 1300, Iris Biotech GmbH, Marktredwitz, Germany) modification of microbeads was accomplished as described previously [25]. All remaining steps were performed as described previously [25]. Data are presented as median MFI and interquartile range (IQR).

### ELISA

CA125 levels in blood samples obtained from patients were measured using the human CA125 ELISA kit (MUC16) SimpleStep (Abcam, LucernaChem AG, Switzerland, #ab195213). ELISA was performed according to manufacture’s protocol.

### Cell culture

Ovarian (IGROV1, A2780, SKOV3, BG1, OVCAR3, OVCAR4, OVCAR5, OVCAR8, OAW42, TYK-nu, KURAMUCHI, OVSAHO, CAOV3, and TOV21G), and breast (T47D and MCF7) cancer cell lines, ovarian surface epithelial cells (HOSE17.1 and HOSE6.3) and normal mammary epithelial cell line MCF10A were grown in RPMI-1640 media (Sigma-Aldrich, Buchs, Switzerland) supplemented with 10% fetal bovine serum (FBS), penicillin (100U/ml) and streptomycin (100 μg/ml) (obtained from Sigma-Aldrich). Fallopian tube secretory epithelial cells (FT237 and FT240) were grown in DMEM-Ham’s F12 medium without HEPES buffer (Sigma) supplemented with Ultroser^Tm^ (PALL corporation, Life Science, Switzerland), penicillin (100U/ml) and streptomycin (100 μg/ml). Another ovarian cancer cell line, EFO27 were grown in RPMI-1640 media supplemented with 20% FBS, penicillin (100U/ml), streptomycin (100 μg/ml) and 1 mM sodium pyruvate (purchased from Sigma). The culture condition for all cell lines were 37 °C in a 95% humidified atmosphere containing 5% CO_2_.

### Flow cytometry

Cells were seeded in T25cm^2^ flask cultivated to 70–80% confluence, washed with PBS, harvested with 1x non-enzymatic cell dissociation solution at 37 °C and counted using trypan blue. Cells were than transferred to 96-well- V-bottom micro test plate (10^5^ cells/well) and pelletized at 400xg for 5 min. Analysis of cell membrane–bound Globo H was performed with two-step staining procedure. Anti-Globo H antibodies (Mbr1_IgM_1:500, diluted in FACSWASH (1% BSA in PBS)) were added to cells, resuspended and incubated on ice for 1 h. After two washes with FACSWASH, cells were incubated with R-PE (1:200) on ice for 30 min. Following two washing steps, cells were incubated with 7-AAD (1:100) on ice for 10 min. After the final wash, cells were resuspended in 200 μl of FACSWASH and analyzed with BD Accuri C6 flow cytometer (BD Pharmingen). Analysis of inhibition experiments using microspheres and human cell lines were analyzed with BD LSR Fortessa. Data analysis was done using FlowJo v10.0.4 (Tree Star Inc., Ashland, USA).

### Statistical analysis

The raw data (obtained as MFI) were log-transformed. Statistical analysis was either performed by GraphPad Prism 6 software or the statistical software R version 3.1.3 (www.R-project.org). Penalized logistic regression and receiver operating characteristics (ROC) analysis was performed using the R packages ‘glmnet’ and ‘ROCR’ [26], respectively. Comparisons between subgroups were performed using Mann Whitney *U*-test. A *p* value < 0.05 was considered significant.

## Results and discussion

Studies in the past suggested that levels of anti-Globo H antibodies are predictive for breast cancer [20, 21]. Recent genomic and transcriptomic analyses by the Cancer Genome Atlas (TCGA) revealed that basal-like breast cancer displays significant molecular similarities to undifferentiated (high-grade) SOC [27, 28]: both diseases share molecular signatures (*e.g.* mutations in *TP53*, *BRCA1/2*, and *RB1*). Thus, we investigated the presence of AGA to Globo H in a cohort of 48 blood plasma samples, consisting of high-grade SOC (*n* = 19) and a control group (*n* = 29) comprising women without or benign disease of the gynaecological tract (*e.g.* leiomyoma, cystadenoma, or fibroadenoma).

**Fig. 1:**
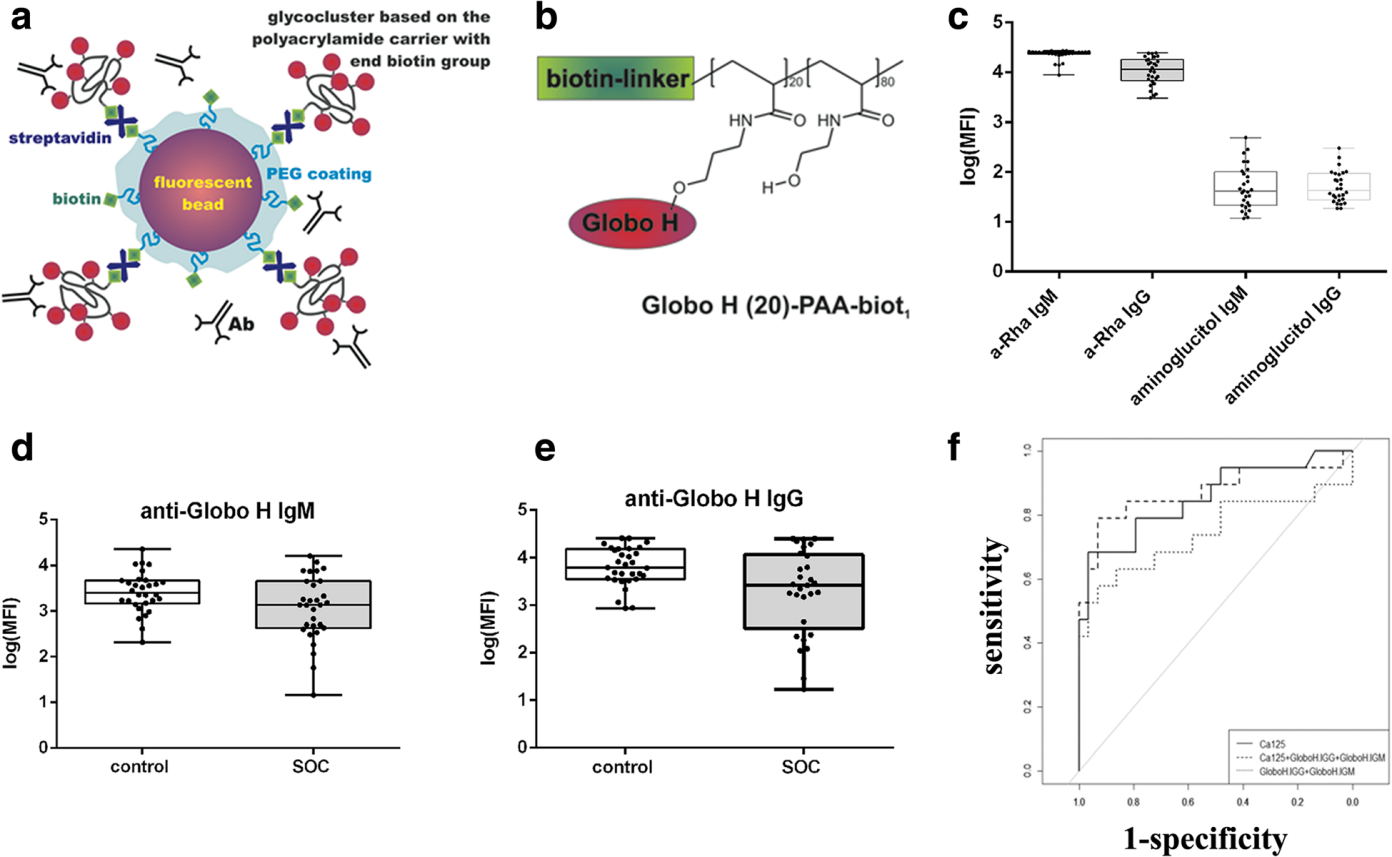
Plasma-derived anti-glycan antibodies of IgG and IgM isotype bind to polymeric presented Globo H glyco-clusters differentiating healthy controls from ovarian cancer comparable to CA125. **a** Schematic presentation of the coupling procedure of end-biotinylated glycoconjugates to streptavidin containing beads. **b** Chemical structure of Globo H conjugated to biotin. **c** Human AGA (log median fluorescence intensity for IgG and IgM separately) binding to positive (alpha-rhamnose) and negative (aminoglucitol) control. (**d** & **e**) Box-and-whisker plot showing distribution of AGA levels directed to Globo H in the blood plasma of serous ovarian cancer (SOC) patients and control group. **f** ROC curve for both antibody classes (IgG and IgM) together and with CA125 together. Individual CA125 levels are displayed

The SGA utilized to measure AGA [22, 25] incorporated coupling of microspheres with an internal fluorescence to chemically synthesized glycans (Fig. 1a) *via* a linker (Fig. 1b). Using monoplexed SGA levels of antibodies to alpha-rhamnose (positive control) and Globo H were generally high for both isotypes in all samples (Fig. 1c-e). In contrast, aminoglucitol (negative control) showed AGA levels below the threshold (Fig. 1c). We observed significantly decreased anti-globo H antibody levels of IgG isotype in SOC patients to the control group (IgG 2642 MFI (338; 6067), *p* = 0.009, Fig. 1d-e), hence clearly discriminating cancer patients from healthy controls. The comparison for the IgM isotype showed a similar trend (1526 (492; 4154), *p* = 0.071, Fig. 1d-e). We next performed ROC analysis including the current clinical ovarian cancer biomarker CA125, which was measured by ELISA during clinical examinations at initial diagnosis. The combination of anti-Globo H IgG and IgM revealed an area under the curve (AUC) of 0.7441. The addition of CA125 in a penalized logistic regression model increased the AUC to 0.8711, which was even higher than CA125 alone (AUC 0.8539). This indicates that anti-globo H antibody levels improve the diagnostic performance of CA125 alone (Fig. 1f).

**Fig. 2:**
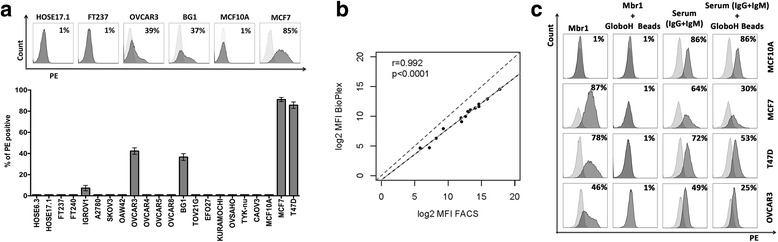
Anti-Globo H antibodies bind ovarian cancer cell lines determined to express naturally synthesized Globo H on the cell surface membrane. **a** Flow cytometry results showing expression of Globo H in selected breast and ovarian cancer cell lines. Representative histograms for cell lines HOSE17.1, FT237, OVCAR3, BG1, MCF10A and MCF7. The percentages of positively stained cells were displayed and are a representative of five independent experiments. Bar chart summarizing five independent experiments on all cell lines tested showing the mean and standard deviation for each cell line. **b** SGA and FACS show high correlation for binding of plasma-derived anti-Globo H antibodies to Globo H-coupled microbeads (SGA) and Globo-H positive cells (FACS, here MCF7 cells); plasma samples 1–14, dilutions 1/40 (SGA) and 1/200 (FACS). MFI values were log-transformed. **c** Flow cytometry data represented as histogram for negative control (*light grey*) and positively stained sample (*dark grey*). The value provided in each histogram refers to the percentage of positively stained cells

Despite this intriguing finding it remained to be elucidated whether anti-Globo H antibodies are capable to bind the native Globo H antigen expressed on ovarian cancer cells and whether plasma-derived AGA also recognize other glycan structures (epitopes similar to Globo H). Thus, we profiled a panel of ovarian (*n* = 15) and breast (*n* = 2, used as positive control, [11]) cancer cells (Fig. 2a) using the monoclonal antibody MBr1 (Fig. 2a). After the evaluation of the MBr1 in breast cancer cell lines MCF7 and T47D, the working concentration 2.5 μg/ml MBr1 was selected. We observed Globo H expression in only three ovarian cancer cell lines, OVCAR3, BG1, and IGROV1 (Fig. 2a). The remaining ovarian cancer cell lines never showed any positivity for Globo H. The normal ovarian surface epithelial cell lines (HOSE17-1 and HOSE6-3), fallopian tube secretory epithelial cells (FT237 and FT240), and the mammary epithelial cell line MCF10A were also negative for Globo H (Fig. 2a). In contrast to our findings, the literature reported Globo H expression also in TOV21G ovarian cancer cells [29]. This discrepancy might be explained by the use of different monoclonal antibodies to Globo H (MBr1 and VK9) in these two studies.

In order to study the binding of naturally occurring anti- Globo H antibodies from patient samples to Globo H-positive ovarian cancer cell lines, we compared SGA and standard flow cytometry. A total of fourteen plasma samples reflecting the spectrum of antibody levels were selected and profiled using both methods. The comparison revealed a high correlation (*r* = 0.992, *p* < 0.0001) concluding that flow cytometry is suitable for AGA profiling (Fig. 2b).

To finally prove that plasma antibodies indeed bind to Globo H on the cell surface, we co-incubated anti-Globo H positive plasma (a pool of five individual samples) with Globo H-coupled microbeads (10^5^ beads). As a negative control we used MCF10A. We found that plasma antibody binding decreased to different extents among all Globo H positive cancer cell lines (MCF7, T47D, and OVCAR3) tested in flow cytometry (Fig. 2c). We did not achieve pronounced decrease of binding using plasma dilution 1/5–1/100 as well as with combination of less than 0.5 × 10^5^ beads. The plasma binding in case of MCF10A was high (86%) and did not decrease after plasma pre-incubation with Globo H beads (Fig. 2c). These data clearly demonstrate that ovarian cancer cells express Globo H and that the antigen is recognized by naturally occurring anti-Globo H antibodies from human blood plasma.

Taken together, we demonstrate that women with SOC and benign disease display different levels of anti-Globo H antibodies. Moreover, these antibodies are capable to bind Globo H expressing ovarian cancer cell lines.

## Conclusions

In this study we report on naturally occurring AGA to a well-known tumour-associated carbohydrate antigen (Globo H) in ovarian cancer. The current study is based on Globo H identified by circulating AGA as well as the monoclonal antibody MBr1. However, future studies have to consider larger cohorts and the measurement of combinations with other representative biomarkers including AGA to evaluate the diagnostic performance of Globo H. Further studies might also incorporate mass spectrometry to confirm the presence of the antigen in ovarian cancer cells. Verification in tissue samples might also support a potential role of Globo H in this malignant disease, *e.g.* by MALDI imaging mass spectrometry [30]. This knowledge might also contribute to our understanding of the functional role of Globo H as it has been associated with cancer stem (−like) cells previously [31].

## Abbreviations

AGA: Anti-glycan antibodies
AUC: Area under the curve
FT: Fallopian tube
FBS: Fetal bovine serum
HOSE: Human ovarian surface epithelium
MFI: Median fluorescence intensity
ROC: Receiver operating characteristics
SOC: Serous ovarian cancer
SGA: Suspension glycan array
TCGA: The cancer genome atlas
